# Exploring the eating disorder curricula of accredited university dietetic programs in Australia and New Zealand

**DOI:** 10.1186/s40337-023-00788-x

**Published:** 2023-04-20

**Authors:** Elizabeth Kumiko Parker, Mellisa Anne Ashley, Deanne Maree Harris, Anita Stefoska-Needham

**Affiliations:** 1 Department of Dietetics and Nutrition, Westmead Hospital, Westmead, NSW 2145 Australia; 2 Sydney School of Health Sciences, Faculty of Medicine and Health, The University of Sydney, Sydney, NSW 2006 Australia; 3 Adult Eating Disorder Service, Western Sydney Local Health District, Sydney, NSW 2145 Australia; 4 Department of Nutrition and Dietetics, Tamworth Rural Referral Hospital, Tamworth, NSW 2340 Australia; 5 Nutrition and Dietetics, School of Medical, Indigenous and Health Sciences, Health Impacts Research Centre, Illawarra Health and Medical Research Institute, University of Wollongong, Wollongong, NSW 2522 Australia

**Keywords:** Eating disorders, Dietitian, Education, Training

## Abstract

**Background:**

Dietitians are viewed as integral members of the multidisciplinary treatment team for people with eating disorders (EDs). However, low levels of perceived confidence, competence, and willingness to practice in this clinical area, have been reported by dietitians and student dietitians. As the extent of ED-specific knowledge and skills-based training within tertiary accredited dietetic programs is currently unknown, this research aimed to: (1) obtain insights into the current ED-specific knowledge base and training content of dietetic curricula in both Australian and New Zealand universities; (2) understand the perspectives of course convenors regarding the role of dietitians in ED treatment and their employment opportunities; and (3) identify gaps and opportunities for improving university programs and the dietetic workforce.

**Methods:**

Course convenors (or their nominated representative) of Australian and New Zealand accredited dietetic programs were invited to participate in a semi-structured virtual interview. A purpose-built question guide was developed to explore the inclusion and/or integration of ED-specific content into the curricula, and the perspectives of course convenors toward the role of dietitians in the treatment of EDs, and their employment opportunities. The interviews were audio recorded, transcribed verbatim, and analysed qualitatively using inductive thematic analysis.

**Results:**

Thirteen participants who represented 14 universities and 19 individual accredited dietetic programs, were interviewed, with some participants representing more than one university. Three dominant themes emerged: (1) varying ED-specific content and training in dietetic programs; (2) unclear dietitian's role in the treatment of EDs, and (3) contrasting views regarding ED clinical practice and employment.

**Conclusions:**

ED-specific content was embedded within all the dietetic programs investigated in this study. However, this content was generally limited to an introductory level, with notable variations found between the depth of content and the type of training provided. Risk-mitigation skill development, such as screening for EDs, and early identification of symptoms, also varied between programs. Therefore, it is recommended that ED-specific skill development and knowledge is enhanced within Australian and New Zealand university programs, to support effective, safe, and timely care for people with EDs. This research has implications for current and future university dietetic program development and the broader dietetic workforce.

**Supplementary Information:**

The online version contains supplementary material available at 10.1186/s40337-023-00788-x.

## Abstract (plainenglishlanguage)

Dietitians are viewed as essential team members in providing care for people with eating disorders (EDs). Previous studies have reported that dietitians and student dietitians perceive lower levels of confidence to work in this area. Appropriate training at university and post-graduation has been identified as one strategy to improve clinician confidence. However, the type and amount of training currently received are unknown. This study aimed to: (1) obtain insights into the current ED-specific knowledge-base and training content of dietetic curricula in both Australian and New Zealand universities; (2) understand the perspectives of course convenors regarding the role of dietitians in ED treatment and their employment opportunities; and (3) identify gaps and opportunities for improving university programs and the dietetic workforce. Thirteen representatives from 14 universities offering 19 different accredited programs were interviewed, and three main themes emerged: (1) varying ED-specific content and training in dietetic programs; (2) unclear dietitian’s role in the treatment of EDs, and (3) contrasting views regarding ED clinical practice and employment. It is recommended that ED-specific skill development and knowledge be enhanced within Australian and New Zealand university programs, to support effective, safe, and timely care for people with EDs.

**Supplementary Information:**

The online version contains supplementary material available at 10.1186/s40337-023-00788-x.

## Background

Dietitians are regarded as experts in food and nutrition, who aim to optimise the nutrition of populations, groups, and individuals, promote health, and prevent and treat disease [1]. In Australia and New Zealand, the dietetic professions have congruent tertiary education and accreditation standards [2], underpinned by national nutrition and professional competency standards [3, 4].  The standards define the skills, attributes, and diligence required by a competent dietitian to practice safely and effectively across a variety of contexts [1], and students are assessed prior to graduation before entering the workforce. However, these competency standards [3, 4] do not include explicit guidance on the role of the dietitian in treating people with specific clinical conditions. Increasingly, this issue has been perceived as a considerable barrier by both dietetic students and qualified dietitians in building their confidence to practice in complex clinical areas, such as eating disorders (EDs) [5].

Additionally, the paucity of information in previous clinical practice guidelines describing dietetic treatment for EDs [6–8], the omission of the role of dietitians in evidence-based treatment manuals for psychological treatment [9–11], and limited research and treatment outcome data in the dietetic ED practice [12–14], have also been identified as impediments to practice in this clinical area. Another potential barrier to practice has been the lack of a clearly defined role for dietitians within the multidisciplinary team for treating people with an ED [5]. In 2020, the Australia & New Zealand Academy for Eating Disorders (ANZAED) published their ED treatment principles and general clinical practice and training standards [15]. Additionally, practice and training standards for dietitians providing ED treatment [16] were also published, to support dietitians to clarify their role within the broader therapeutic team, to understand the minimum performance requirements to treat individuals with an ED, and to clarify the expectations required for ED-specific training and clinical supervision. However, it is unknown if these training standards [15, 16], have enhanced the confidence and competence levels of dietitians working with people with EDs or have been effectively translated into clinical practice.

Perceptions of inadequate ED-specific knowledge and skills-based training in university programs have also been reported by dietetic students and practising dietitians as contributing factors to their reluctance to practice clinically with people with an ED [5]. However, the extent to which these elements of ED-specific knowledge and skills-based training are included in the dietetic university curricula in Australia and New Zealand are currently unknown. Therefore, an exploratory research approach was used to conduct semi-structured interviews with university program convenors to (1) obtain insights into the current ED-specific knowledge and skills-based training of dietetic curricula in both Australian and New Zealand universities; (2) understand the perspectives of course convenors regarding the role of dietitians in ED treatment and their employment opportunities; and (3) identify gaps and opportunities for improving university programs and the dietetic workforce. This study has potential implications for improvements to current and future accredited university dietetic programs, and the broader dietetic workforce.

## Methods

### Study design

This qualitative descriptive study drew upon an interpretive phenomenological approach [17] to capture participants’ thoughts and feelings regarding the topic in everyday terms, using individual interviews. An exploratory interview approach was used rather than an audit of curricula content, to facilitate open discussion with participants. The reporting of findings adhered to the guidelines outlined in: The Consolidated Criteria for Reporting Qualitative Research (COREQ) [18] (Additional file 1). This study received ethics approval from the Wollongong University Human Ethics Committee (2019/057).

### Participants and recruitment

Accredited dietetic programs offered by universities in Australia and New Zealand were identified from the Dietitians Australia and New Zealand Dietitians websites [1, 19], respectively. These university dietetic programs were investigated, to explore the ED-specific content of curricula of close nations that are both represented through the Council of Deans of Nutrition and Dietetics [2], and have reciprocating recognition with respect to their professional dietetic associations [20]. Furthermore, documents such as the ANZAED ED treatment principles and general clinical practice and training standards [15] and practice and training standards for dietitians providing ED treatment [16], and the Royal Australian and New Zealand College of Psychiatrists clinical practice guidelines for the treatment of EDs [6], apply to both nations. Eighteen universities were identified, collectively offering 23 accredited dietetic programs. Of these 23 programs, there were 15 postgraduate Master degrees and 8 undergraduate Bachelor degrees (five of which were Bachelor degrees with Honours); or alternatively classified as levels 9, 8, and 7 respectively, according to the Australian Qualifications Framework [21]. Course convenors were invited to participate via email and could nominate an alternative person if they were unable to participate. Informed written consent was obtained from each participant prior to completing the interview.

### Data collection

A purpose-built question guide was developed by the authors for use in the interviews to explore the inclusion and/or integration of ED-specific content into the accredited dietetic programs in Australia and New Zealand (Additional file 2). Some questions were designed to probe the perspectives of course convenors regarding the role of dietitians in ED treatment and their employment opportunities. The content validity of the question guide was addressed through the researchers' experience with dietetics course delivery and/or familiarity with the clinical area and guided by a previous study investigating disability-related content taught in accredited dietetic programs [22]. The collective knowledge and expertise embedded within the research team enabled meaningful content validity of the question guide so that it was relevant in relation to the project’s intent.

Participants engaged in a semi-structured, virtual interview using Zoom™ (Version 3.3, 2019) [23], a cloud-based video-conferencing package, between March 2019 and March 2021. Interviews were facilitated by one member of the research team and lasted between 30 and 45 min each. All interviews were audio recorded and transcribed verbatim using Otter.ai™ (2021). There were no repeat interviews required.

### Data analysis

All interview transcripts were compared to the original audio recordings for accuracy. Transcripts were analysed inductively using Braun and Clarke’s six phases of thematic analysis [24]. Three researchers (EP, MA, DH) independently read and familiarised themselves with the transcripts and manually coded the data. These researchers contributed to the discussion of codes and emerging themes until consensus agreement was reached on dominant themes. To establish the rigour of the study, the criteria proposed by Lincoln and Guba was used [25]. Confirmability was ensured through independent coding by multiple reviewers and a clear audit trail. Transferability was demonstrated by detailed (thick) descriptions of participants and the context [26], and credibility was demonstrated with the use of verbatim participant quotes.

## Results

Thirteen participants (11 females, 2 males) representing 12 universities in Australia and two universities in New Zealand responded (response rate 78%), and they represented 19 out of a possible 23 (83%) accredited dietetic programs, including Master degree (n = 12), Bachelor degree (n = 2) and Bachelor with Honours degree (n = 5). Of the 13 participants, eight were course convenors involved in program design and quality assurance; three participants were university academic staff who delivered the ED-specific content and worked as a clinician treating people with EDs, and two participants were external clinicians, treating people with EDs, who delivered the ED-specific content as guest lecturers. One of the external guest lecturers provided the ED-specific content across multiple university programs. All participants who provided consent were interviewed, and no participants dropped out during the study period.

Analysis of the data yielded three dominant themes, including (1) varying ED-specific content and training in dietetic programs; (2) unclear dietitians’ role in the treatment of EDs, and (3) contrasting views regarding ED clinical practice and employment. The main themes are summarised alongside key characterising statements (Table 1).

**Table 1:** Main themes and characterising statements

Themes	Characterising statements for each theme
Varying ED-specific content and training in dietetic programs	Programs differ in the type/volume of ED-specific content, the degree of facilitation of ED-specific skill development, and the types of teaching approaches utilised
	Curricula space is limited
	﻿ED-specific practical placement opportunities are sparse
Unclear dietitian’s role in the treatment of EDs	Limited published guidance regarding the role of the dietitian in the treatment of EDs
	Lack of data on dietitian treatment outcomes
	Confusion regarding boundaries/scope of practice
Contrasting views regarding ED clinical practice and employment	Some participants expressed that new graduate dietitians are not likely to practice in EDs as it is a specialist clinical area
	Other participants felt new graduates are very likely to encounter a person with an ED due to growing employment opportunities in private practice

### Varying ED-specific content and training in dietetic programs

All participants identified the inclusion of ED-specific content within their program curricula, however, several between-program differences emerged. Differences in the depth of ED-specific information, the skill-development strategies utilised, and the prioritising of content were identified by participants. ED-specific lectures were limited to a one two-hour lecture inclusive of a case study presentation for most curricula (Table 2).

**Table 2:** Time allocated for ED-specific lectures in dietetic curricula

Allotted ED-specific lecture time	Number of dietetic programs
1-h lecture	1
2-h lecture	9
3-h lecture	3
4-h lecture with workshop	1

These lectures were delivered within a broader medical nutrition therapy module or program unit. Many participants reported that the ED lecture was at an introductory level, describing an overview of the diagnostic criteria for EDs, with an emphasis on the development of nutrition assessment skills, rather than the provision of dietetic approaches in the treatment of EDs, such as therapeutic engagement, nutrition counselling skills, and ED-specific treatment modalities, for example:

*Students need skills to learn management techniques and explore the evidence, for any and every condition that comes along, as well as the nutritional care process, and be able to apply that to whatever the disease state might be, rather than spend a lot of time on loads and loads of individual diseases. And thinking about where students are likely to go on placement as well and what sort of clients, they’re likely to see, most of them aren’t going to see eating disorder patients unless they’re acutely unwell (Participant 3).*

Pre-reading activities are typically incorporated in university programs to cover content that is unable to be included in the allocated lecture time. Pre-reading was mentioned by a few participants in this study as a strategy for including more ED-specific content in the curricula. Additionally, the inclusion and depth of risk-mitigation strategies, such as skills development for screening for EDs and/or disordered eating varied. For example:

*[The ED lecture] is usually 2 hours. Pre-reading is diagnostic criteria. I'll usually do around about 20 minutes on understanding eating disorders, using various models. We then have a look at the role of the dietitian, in terms of screening for eating disorders. So even if you're not working in eating disorders there is still a role in terms of identifying and screening… So, we do probably about 40 minutes on inpatient treatment. And that includes an element of refeeding syndrome. And we probably do about half an hour on outpatient care. And then we do around about 10 minutes on referral pathways and relevant stakeholders and services like Butterfly Foundation (Participant 11).*

ED-specific experiential training opportunities were rare across all dietetic university programs investigated in this study. Generalised skill training was more common, such as introductory dietary counselling, motivational interviewing techniques, core meal plan development skills, managing people at risk of refeeding syndrome, and the Health At Every Size® (HAES®) [27] approach, with the inclusion of some practical teaching sessions. The utilisation of simulations using paid actors, or the development of videos demonstrating dietetic counselling skills and psychoeducational material provided in treatment, was suggested by participants as a valuable teaching resource to address the paucity of experiential ED-specific training opportunities. For example:

*I wouldn't mind having a specific [simulation] activity for eating disorders, but it's just so hard to fit everything in. You are trying to sort of balance what they need for placement to pass placement successfully, but also what they need for their future (Participant 10).*

ED-specific practical placements were rare across all university programs. Seven participants reported their program was not affiliated with a placement partner with access to a specialist ED service, four participants reported their placement partners did include access to a specialist ED service, although student exposure to this service was not guaranteed during their placement, and two participants were unable to comment as they were not involved in coordinating student practical placements. Unplanned interaction with a person with an ED may occur, during practical placement, but any interaction was primarily within an observational capacity. For example:

*[Clinical Placement exposure to eating disorders is] only from an observational perspective. Yeah, so we don't find that most of the placement sites have dietitians that have eating disorders as part of their current caseload, so they're not all really kind of confident in taking students in eating disorders and allowing students to take on any sort of patients…. it would just be within the hospitals, like a general medical ward kind of thing. Yeah, and they are terribly unwell usually if they are on a gen med ward. Yeah, and quite a complicated patient for those students to take on (Participant 13).*

The lack of ED-specific practical placement opportunities for students was attributed to limited ED service providers by placement partners. Additionally, public ED services were reported to frequently decline student involvement. Perceptions of high staff turnover, and thereby a lack of committed supervision, and clinicians (including dietitians) prohibiting student involvement due to their own lack of ED experience were cited. For example:

*The students would love it, but there actually is so few eating disorders clinics in the public sector…. There's some community, outpatient support, but mostly it falls on private practice. Yeah, so, you know, we just don't send students to private practice, we would love to, but for the most part, the dietitians are just not interested in having a student. So, for those reasons, we just don't send students to eating disorder placements (Participant 12).*

*If there are placements, it's not regular, and then because of the change in, at least locally, there's a high turnover of staff in eating disorders. So that's been part of the problem (Participant 7).*

### Unclear dietitian’s role in the treatment of EDs

Most participants acknowledged that their dietetic program contained limited content on ED-specific dietetic treatment approaches, due to the limited published guidance on the role of the dietitian in the treatment of EDs, the varied treatment approaches that can be utilised, and the paucity of dietetic outcome data. For example:

*I think there’s a need for more research in that area, to necessitate the need for dietitians. It’s a weird place to work in, I think for dietitians all over the world, because evidence-based treatment doesn’t incorporate dietitians. And so anywhere that uses an evidence-based treatment is going to be going off the book if they use a dietitian…there is a tension there in the field between how much dietitians can do, and how much should they do? And a lot of people think that dietitians shouldn’t be involved in teams and in care (Participant 2).*

Identified by study participants as gaps in current program content, were scope of practice (to highlight what tasks and activities were permissible in practice), reflective practice (to assist dietitians to assess their own strengths and limitations in knowledge and experience, and to guide future changes to practice), and risk mitigation strategies (to assist in decisions about when to seek professional assistance and when to refer to more experienced clinicians). For example:

*I would hope that people don't just go straight into it [working in eating disorders] without having adequate experience and training and supervision and support. And as I think dietitians without adequate experience, all those things could get you into some hot water, and basically, potentially do more harm than good. Yeah, and for themselves, not just for the patients (Participant 8).*

Participants generally agreed that these elements required greater emphasis in curricula, alongside the existing National Nutrition Competency Standards [3, 4], to promote safe, effective practice when working with people with EDs. The inappropriateness of dietitians managing clients with EDs if they themselves were struggling with the signs and symptoms of an ED was also of concern to some participants. For example:

*There is a proportion of dietetics students who do have eating disorders themselves, so we have this chat about that right at the beginning before we start any practice… They are not to practice if they’re unwell’ (Participant 3).*

Participants highlighted that course lecturers generally advised their students to enrol in post-graduate training and clinical supervision to work in a specialised clinical area, such as EDs. However, the specific details on how and where to access further training and clinical supervision were not always shared. For example:

*We make sure that the dietitian who gives that lecture does say…. this is a specialist area so it’s not expected that you would finish your course and go out and start treating eating disorder patients straight away. You need to get some more training, this is how you might want to find a mentor in that area, and this is how you might want to get some support in that area. So, we certainly make sure that that comes across in that lecture (Participant 1).*

*Eating disorder treatment is still something I think we battle with [by] not having a clearly defined role in terms of evidence-based practice models….it can be an area where, as a new grad, you can invest too much time and become overly involved. So, I think boundaries are important as well…. I think having DA [Dietitians Australia] support mandatory supervision would be helpful… it doesn't matter how much experience you've got in the space; supervision is always valuable. And I think that without the DA supporting it because it comes at a cost, people sort of see it as a bit of a barrier. So, I think people might be a little bit safer in their practice but also feel a bit more confident if they've got something like supervision in place (Participant 11).*

### Contrasting views regarding ED clinical practice and employment

Some study participants expressed a belief that newly graduated dietitians are unlikely to encounter a person with an ED in the workplace, thus the necessity for ED-specific training, beyond an introductory level, was not a priority within the curriculum. Furthermore, a subset of participants in this study regarded EDs as a specialist area and therefore assumed that new graduates would not be employed to work with people with EDs, specifically in the public health system or in specialised ED services directly following university. As a result, some participants stated that the intention of ED-specific knowledge and training at university was only to introduce the clinical area, with additional resources and support for any ED-specific post-graduate education, training, and clinical supervision. For example:

*I think the truth is that very few dietitians who will end up working in eating disorder treatment, it’s a very specialist area and you know it’s just one of many areas the dietitians may or may not work. So, I don’t necessarily think that we need to do more on eating disorder treatment. You must learn once you get out there and I don’t know if there is a point in learning more before you do (Participant 2).*

*Very limited [eating disorder employment opportunities] in the public sector. In the private sector, I think there's more opportunity. It's whether those opportunities are supported by appropriate wraparound supporting safe and ethical practice in terms of supervision and mentoring, that type of thing (Participant 11).*

In contrast, other study participants acknowledged that new graduates are increasingly working in generalised private practice settings after university, and are therefore more likely to encounter a person with an ED, including an undiagnosed or sub-clinical ED. A small number of participants stated the importance of developing skills in screening for EDs, boundary-setting, conducting comprehensive nutrition assessments, and identification of appropriate referral pathways, during students’ university training. For example:

*There’s a reasonable proportion of students who are moving into private practice, that seems to be where the work opportunities are...and I think probably more likely to come across undiagnosed eating disorders… I think the main message is that if students are going to be exposed to eating disorders in practice that they should do further training and they should get a mentor….I think we would give them the skills to be able to identify when they may be dealing with a patient who does have an underlying eating disorder, so that’s probably the main skill they would come away with is recognising some of the features of those….And either referring on if they didn’t have the skills to manage that situation or if they’re likely to be coming across that regularly yeah as I said get a mentor and do some further training…. I think they could do a nutrition assessment. I think they would struggle with providing an intervention (Participant 5).*

*I wouldn't expect new grads to take that [people with eating disorders] on in private practice, but they'll certainly be exposed to it (Participant 13).*

## Discussion

This study explored the ED-specific content and training of accredited dietetic curricula in Australia and New Zealand, from the perspectives of course convenors and/or lecturers. ED-specific content was embedded in all the dietetic programs investigated in this study. However notable variations were found between programs, reflected by differences in the time allocated for the ED-specific content, the depth of the ED-specific knowledge provided, and the skill-development strategies employed. Furthermore, there were contrasting perceptions between course convenors in relation to the priority and purpose of ED-specific knowledge and skill set for their students. Additionally, this study revealed limited ED-specific practical placement opportunities by providers who partner with universities. These inconsistencies regarding the depth of ED-specific content provided, the time allotted for ED-specific content within the current university curricula, and the limitations found in opportunities for both experiential skill-development and practical placement opportunities, are problematic, as both students and new-graduate dietitians in Australia have reported low confidence levels to work with people with an ED [28]. Similarly, practising dietitians in the United States [29, 30], have reported lower levels of confidence in counselling people in this clinical area. As improvements in people’s symptoms are highly correlated with their therapists’ perceptions of confidence [31], treatment outcomes for this vulnerable patient group may be compromised, if clinicians exhibit lower levels of confidence to practice in this clinical area.

Course design in accredited dietetic programs is guided by the National Competency Standards for Dietitians in Australia [3] and Professional Competencies & Standards for Dietitians in New Zealand [4]. Using these standards, students in their final year of study are assessed for competency and safe practice in the broader clinical context, rather than in specific clinical areas. Our findings indicate that the ED-specific content described in the university curricula was generally limited to an introductory level, frequently consisting of a 2-h lecture with a case study and covering an overview of the diagnostic criteria of EDs, with prioritisation of developing nutrition assessment skills. These findings are consistent with earlier work by Trammell et al. [29] who have also reported that ED-specific lectures in the United States were limited to a brief introduction to EDs, with little information on dietetic treatment approaches [29]. In Australia, the authors have previously reported that both practising dietitians and final year dietetic students perceive the current ED-specific education and training at universities to be inadequate [5]. In this previous study [5], the authors recommended augmenting the ED-specific education and training components within all university programs, beyond an introductory level, to enhance the preparedness of dietitians to practice in this clinical area and reduce their general reluctance to treat patients with an ED. If reluctance to treat patients with an ED continues within the dietetic profession, accordingly, the rate of unmet treatment demands is likely to increase [32, 33], potentially reducing opportunities for the early detection of an ED [29]. This is problematic, as early detection and intervention have been associated with improving treatment outcomes and reducing illness severity and duration [34].

Participants also reported that students received additional training in core, generalised, dietetic skills outside of the ED-specific teaching session. This included generalised dietary counselling techniques, motivational interviewing strategies, meal plan development, managing patients at risk of refeeding syndrome, and the HAES® treatment approach. Interestingly, more than half of final-year students and new graduate dietitians in the study published by Denman et al. [28], reported lacking confidence in applying treatment approaches such as motivational interviewing strategies and the HAES® approach to patient care. This may be expected as motivational interviewing is a very specific approach to addressing motivation and behaviour change, and is likely to require multiple hours of practical training to demonstrate competence in utilising this technique. Furthermore, despite the emerging recognition of the HAES® approach in the treatment of people with binge eating disorder [35], HAES® is not an evidence-based treatment approach for EDs. In Canada, Cairns et al. [36] reported 71% of surveyed dietitians (n = 65) were dissatisfied with the education opportunities available to dietitians in EDs, particularly nutrition counselling [36].

Of the university programs investigated, few provided content on risk-mitigation strategies such as effective screening skills for EDs, and appropriate ED referral pathways. The study participants who prioritised these learning strategies for their students were influenced by two key factors. Firstly, recognition of the increasing prevalence of EDs and disordered eating behaviours globally [37], and secondly, the increasing rates of new graduate dietitians employed within multiple and diverse workplace settings, with the most common location being generalised private practice settings in Australia [38, 39]. Collectively, these factors contribute to the increased likelihood of an emerging dietitian encountering an undiagnosed patient with an ED [28], as many patients seek treatment of symptoms for their ED, even in the absence of a formal ED diagnosis [32, 40, 41]. For this reason, dietitians have been described as ‘first responders’ in EDs and can play an important role in the identification of EDs, thereby facilitating early access to treatment and early intervention when appropriately trained [42]. Health professionals who have limited knowledge and clinical experience in the recognition of ED symptoms, signs, and referral pathways, may unintentionally cause harm, including delays in patient recovery [43]. Therefore, it is essential that any dietitian planning to practice clinically after graduation, has knowledge of these risk-mitigation strategies, alongside reflective practice skills, and familiarity with a defined scope of practice such as the ANZAED practice and training standards for dietitians [16]. Currently, the National Nutrition Competency Standards [3, 4] stipulate that an entry-level dietitian can acknowledge their personal limitations in professional knowledge and skill-set, as part of demonstrating safe practice.

In addition to developing skills in risk mitigation, the understanding of professional boundaries and learning boundary-setting skills, are essential for dietitians working within any clinical setting. Notably, some participants in this study reported their concerns regarding the prevalence of eating disorders among dietetic students. These concerns have been supported by studies demonstrating 14–30% of students surveyed in food-related degrees (including nutrition and dietetic students) report symptomology consistent with disordered eating or exercise addiction [44–46]. Boundary-setting skills can facilitate safe and sustainable practice by addressing the scope of practice, treatment boundaries, self-disclosure, confidentiality, dual relationships, time management, and personal boundaries [47].

Clinical supervision also plays an important role in facilitating dietitians to upskill in EDs [48, 49], and has been highlighted in the standards of practice for dietitians working in EDs [16], as well as the recently launched ANZAED Eating Disorder Credential [50]. Furthermore, clinical supervision after graduation, and throughout a dietitian’s working life, plays a key role in building clinician confidence, competence, guiding safe practice, developing communication skills, and improving clinician satisfaction [51, 52]. Notably, many participants in this study advised their students to take part in a clinical supervision arrangement before beginning employment in clinical ED practice. However, engagement in clinical supervision is voluntary. Access issues to dietetic clinical supervision in EDs have been reported, with barriers including high cost, balancing competing priorities with increasing workloads, and limited availability of trained supervisors, particularly in rural and remote settings [5]. Therefore, an online offering of facilitated, peer group supervision for dietitians working in EDs, may be one solution. The online group clinical supervision model utilised by Davis et al*.* [49], was found to deliver an accessible, acceptable, and effective resource to dietitians in both public and private settings across a large geographical area. Ninety-eight percent of respondents reported positive changes to clinical practice, including increased self-assessed confidence to implement evidence-informed guidelines, improved engagement with patients with EDs, and advocacy for patients. Other benefits reported by respondents, included increased support in their clinical work, improved coping strategies for working with the physical and mental health complexities of patients with EDs, and greater enjoyment working within this clinical area [49]. Mandated clinical supervision hours by professional organisations may be one ongoing strategy to stimulate the uptake of formalised clinical supervision arrangements.

Specific guidelines for the outpatient dietetic treatment of EDs [53], as well as standards of practice for dietitians working in EDs [16], have only been published recently, therefore, historically, there has been an absence of evidenced-based nutrition approaches to treating EDs. As a result of this gap, participants expressed difficulty incorporating detailed information on dietetic treatment approaches for EDs into university curricula. These findings are consistent with Hart et al. [54] who reported a lack of standard nutritional management for anorexia nervosa and bulimia nervosa among dietitians working in the clinical management of patients with EDs in Australia [54]. More recently, a systematic review by Yang et al*.* [55] revealed mixed results with respect to the effectiveness of including a dietitian in outpatient ED treatment, due to inconsistent descriptions of what a dietetic intervention entails [55].

The recently published ANZAED practice and training standards for dietitians providing ED treatment [16], the Dietitians Australia ED Role Statement [56], the consensus-based guidelines for outpatient dietitians [53], as well as the NEDC Dietitian Decision-Making Tool [57], are available, to support dietitians in providing effective and safe care. The latter document is specifically targeted toward dietitians in the community or private practice and provides a step-by-step guide to support dietitians in the identification of, and response to, patients with EDs [57]. Additionally, a newly published overview of the role of the dietitian in the treatment of EDs [42], lists nutrition frameworks that can be utilised as practical tools to deliver nutrition interventions for EDs, including RAVES [58], The REAL Food Guide [59], the rule of threes [60], and Plate-by-Plate Approach [61]; and outlines core patient nutrition education topics targeted during nutrition intervention for EDs [42]. However, unless these documents are acknowledged or included by universities in the planning and delivery of the ED-specific content, then students are likely to be unaware of their value in dietetic practice. Future research is recommended, to assess if these documents are referenced in dietetic curricula, and to understand the impact of these documents, on the perceived levels of confidence, competence, risk mitigation skills, and clinician readiness to provide dietetic treatment for patients with EDs.

Participants reported that ED-specific practical placement opportunities were rare and were mostly experienced by students in an observational capacity, typically occurring on a few occasions when ED services were provided by the placement partner. These findings are consistent with the authors’ previous focus group study of dietitians and final-year dietetic students [5]. The importance of enhancing theoretical knowledge with practical learning opportunities to promote skill development and build confidence has been previously reported [5, 30]. With the lack of practical placement opportunities in EDs unlikely to change in the near future, alternative training approaches such as simulations with paid actors, or access to recorded vignettes of mock consultations using various dietetic treatment approaches in EDs, require consideration. Virtual reality simulations may be an additional consideration, as this type of training has been used in other health professional training for EDs (for example psychology students) and includes diagnostic interviews [62].

Postgraduate training opportunities appear to be an additional pathway to equip dietitians with the skills and confidence to treat patients with an ED effectively, in combination with clinical supervision. While participants frequently reported their lecture content highlighted the need for further training and clinical supervision to work in a specialised clinical area, including EDs, the specifics of what that further training should encompass and where to access this information were undefined. These findings are consistent with the authors’ previous research, which found dietitians and dietetic students are seeking a framework to guide them for ongoing post-graduate ED-specific professional development [5]. The recently launched ANZAED Eating Disorder Credential may address this gap [50]. This credential, is a formalised recognition of the minimum qualifications, knowledge, training, and professional development required, to deliver a minimum standard of safe and effective care to individuals with EDs [50].

While this current study provided insights into the ED-specific content of several accredited dietetic programs in Australia and New Zealand, one limitation of this study was the variation found in the specificity of information provided by the participants. This was likely due to the inclusion of both course convenors who were involved in the overall design of the program curricula and not in the delivery of the ED lecture content themselves, as well as the inclusion of ED-specific lecturers, who developed and provided the ED content within an allocated time frame. Four of 18 universities offering accredited dietetic programs across Australia and New Zealand were not represented in our study sample, potentially limiting the full scope of exploration of perspectives. However, participation in this study was representative geographically, as most states/ territories in Australia and regions in New Zealand were included.

## Conclusions

This study revealed notable variations in the ED-specific content of accredited dietetic programs in Australia and New Zealand. Student dietitians are provided with the training and skills to meet the Dietitians Australia National Competency Standards [3] or New Zealand Dietitians Board Professional Standards and Competencies [4], which do not specify explicit standards for individual clinical areas such as EDs. The need for further ED-specific training and clinical supervision was voiced by course convenors and lecturers, however, little description of what this entails was offered. Many participants in this study felt that student dietitians and emerging dietitians are unlikely to work in this clinical area after graduation, despite workforce data suggesting that dietitians are more likely to encounter patients with an undiagnosed ED within any clinical setting, notably to seek treatment for their symptom management, even in the absence of a formalised ED diagnosis [32, 40, 41].

Student dietitians, emerging dietitians, and experienced clinical dietitians in the workforce have identified several gaps in the ED-specific knowledge and training at universities in Australia [5, 28], and that addressing these deficits would assist in improving their preparedness, confidence, and perceived competence to work with patients within any clinical setting, and importantly, where a patient with an ED or undiagnosed ED may be encountered [5]. Therefore, enhancement of ED-specific information and skill development at Australian and New Zealand universities is highly recommended, to support effective, safe, and timely care for patients with EDs. This research has potential implications for future university dietetic program development and redesign, as well as the broader dietetic workforce.

## Supplementary Information

**Additional file 1**: COREQ Checklist.

**Additional file 2**: Interview Question Guide.

## Data Availability

The datasets used and/or analysed during the current study are available from the corresponding author on reasonable request.
