# Supplementary material for: Exploring the eating disorder curricula of accredited university dietetic programs in Australia and New Zealand
Source: J Eat Disord. 2023 Apr 20;11:63. doi: 10.1186/s40337-023-00788-x (PMC10116702; doi:10.1186/s40337-023-00788-x)
Supplement: Supplementary file 2 — Additional file 2: Interview Question Guide. [file 40337_2023_788_MOESM2_ESM.docx]

Supplement 2

**Interview Question Guide**

1. How is the topic of eating disorders incorporated into the teaching curriculum?
2. Can you explain what kind of subject this topic would be covered in (e.g., clinical, or therapeutic dietetics? A counselling subject or a community and public health subject?
3. Do you teach this as a separate topic specifically on eating disorders or is it part of a larger module (e.g., mental health)?
4. Does your program currently incorporate placement or work integrated learning opportunities in eating disorders?

a.     If yes, what are they and how much time does the student spend in that area?

b.    If yes, are these undertaken by all students? Randomly allocated or a specific request.

1. Do you have any plans to change learning activities or placements in eating disorders over the next 12 months?

a.     If yes, in what way?

b.     Why?

c.     Are there any aspects of the current curriculum that you feel need improvement?

1. What do you perceive to be the role of the dietitian in eating disorder management?
2. Do you think that is changing over time? If yes how?
3. In terms of employment opportunities for your students, how do you see eating disorder treatment?
4. How do you think you could better prepare students for working in eating disorders?
5. Would you be willing to share relevant course material?
6. Is there anything else you wanted to add?
